# Antibiotic Prescribing Patterns for Respiratory Tract Illnesses Following the Conclusion of an Education and Feedback Intervention in Primary Care

**DOI:** 10.1093/cid/ciad754

**Published:** 2024-01-25

**Authors:** James J Harrigan, Keith W Hamilton, Leigh Cressman, Warren B Bilker, Kathleen O Degnan, Michael Z David, David Tran, David A Pegues, Lauren Dutcher

**Affiliations:** Division of Infectious Diseases, Department of Medicine, University of Pennsylvania Perelman School of Medicine, Philadelphia, Pennsylvania, USA; Division of Infectious Diseases, Department of Medicine, University of Pennsylvania Perelman School of Medicine, Philadelphia, Pennsylvania, USA; Department of Biostatistics, Epidemiology, and Informatics, University of Pennsylvania Perelman School of Medicine, Philadelphia, Pennsylvania, USA; Department of Biostatistics, Epidemiology, and Informatics, University of Pennsylvania Perelman School of Medicine, Philadelphia, Pennsylvania, USA; Division of Infectious Diseases, Department of Medicine, University of Pennsylvania Perelman School of Medicine, Philadelphia, Pennsylvania, USA; Division of Infectious Diseases, Department of Medicine, University of Pennsylvania Perelman School of Medicine, Philadelphia, Pennsylvania, USA; Independent Researcher; Division of Infectious Diseases, Department of Medicine, University of Pennsylvania Perelman School of Medicine, Philadelphia, Pennsylvania, USA; Division of Infectious Diseases, Department of Medicine, University of Pennsylvania Perelman School of Medicine, Philadelphia, Pennsylvania, USA

**Keywords:** antibiotic stewardship, antimicrobial stewardship, respiratory tract infections, primary care, antibiotic prescribing

## Abstract

**Background:**

A study previously conducted in primary care practices found that implementation of an educational session and peer comparison feedback was associated with reduced antibiotic prescribing for respiratory tract diagnoses (RTDs). Here, we assess the long-term effects of this intervention on antibiotic prescribing following cessation of feedback.

**Methods:**

RTD encounters were grouped into tiers based on antibiotic prescribing appropriateness: tier 1, almost always indicated; tier 2, possibly indicated; and tier 3, rarely indicated. A χ^2^ test was used to compare prescribing between 3 time periods: pre-intervention, intervention, and post-intervention (14 months following cessation of feedback). A mixed-effects multivariable logistic regression analysis was performed to assess the association between period and prescribing.

**Results:**

We analyzed 260 900 RTD encounters from 29 practices. Antibiotic prescribing was more frequent in the post-intervention period than in the intervention period (28.9% vs 23.0%, *P* < .001) but remained lower than the 35.2% pre-intervention rate (*P* < .001). In multivariable analysis, the odds of prescribing were higher in the post-intervention period than the intervention period for tier 2 (odds ratio [OR], 1.19; 95% confidence interval [CI]: 1.10–1.30; *P* < .05) and tier 3 (OR, 1.20; 95% CI: 1.12–1.30) indications but was lower compared to the pre-intervention period for each tier (OR, 0.66; 95% CI: 0.59–0.73 tier 2; OR, 0.68; 95% CI: 0.61–0.75 tier 3).

**Conclusions:**

The intervention effects appeared to last beyond the intervention period. However, without ongoing provider feedback, there was a trend toward increased prescribing. Future studies are needed to determine optimal strategies to sustain intervention effects.

## Teaser

Cessation of a previously implemented education and feedback program focused on improving antibiotic prescribing for respiratory tract infections was associated with a moderately sustained reduction in the rate of antibiotic prescribing in primary care.

**(See the Editorial Commentary by Dumkow on pages 1128–30.)**

Inappropriate ambulatory antibiotic prescribing represents an ongoing challenge for antibiotic stewardship programs. Factors that contribute to this include heterogeneous patient settings, lack of rapid diagnostics for some common infections, and ingrained practices and attitudes toward antibiotic prescribing [1–3]. It is estimated that 59.1% of antibiotic expenditures occur in nonhospital settings. In 2021, there were approximately 636 outpatient antibiotic courses for every 1000 persons in the United States [4, 5]. Excessive antibiotic use carries increased financial costs to patients, side effects including *Clostridioides difficile* infection, and risks of subsequent infections with drug-resistant organisms [6, 7]. Respiratory tract diagnoses (RTDs) represent a significant portion of outpatient antibiotic prescribing, particularly in primary care, and antibiotic prescribing for RTDs is frequently unnecessary [8, 9]. While outpatient stewardship interventions have been associated with decreased rates of antibiotic prescribing for RTDs, there is limited evidence on the long-term post-completion effects of such interventions [3, 10–14], which has implications for the generalizability and maintenance of outpatient stewardship programs. In this study, we assess long-term durability after the conclusion of a successful time-limited, RTD-focused education and peer-comparator feedback intervention on the rates of antibiotic prescribing for RTDs in primary care [15].

## METHODS

### Study Design and Population

We previously conducted a stepped-wedge cluster randomized trial in 31 primary care (PC) clinics in the University of Pennsylvania Health System (UPHS), the results of which have been described [15]. In this previous study, practices were randomized into 6 clusters. For the first 6 months of the intervention (1 October 2017 through 31 March 2018), on a rolling basis, clinics in each cluster received a 1-time educational session along with clinic resources focused on appropriate antibiotic prescribing for common RTDs. Starting the month after this educational session, prescribers in each clinic were given monthly electronic feedback on their rates of prescribing with peer comparison through 31 October 2018, at which point funding for the intervention concluded and feedback was ceased.

For the study described here, we included the same PC clinics and prescribers that were included in the initial study. Resident physicians as well as other prescribers who joined the participating practices after the start of the intervention (and thus were not included in the initial analysis) were excluded. All encounters from 1 July 2016 through 31 December 2019 were included. We defined the post-intervention period (the period following cessation of peer comparison feedback reports) as encounters that occurred between 1 November 2018 and 31 December 2019, concluding prior to the onset of the coronavirus disease 2019 (COVID-19) pandemic. We included the same pre-intervention and intervention periods (the comparison periods) of the initial trial, which consisted of a pre-intervention period starting 1 July 2016 up until the month each cluster was given the education intervention, and an intervention period beginning the month after each cluster received the education intervention continuing through 31 October 2018.

### Data Collection

Patient demographic data, antibiotic prescribing data, and *International Classification of Diseases, Tenth Revision, Clinical Modification* (ICD-10-CM), codes were abstracted from a data warehouse that extracts all data from the electronic health record. Charlson comorbidity indices for each encounter were calculated using ICD-10-CM codes from the index visit and previous 6 months using a method previously described [16]. Prescriber and practice demographic data from the original intervention study data were used [15].

### Outcome Assessment and Definitions

The primary outcome was the presence of an antibiotic prescription during an in-person RTD visit. Analyses were performed at the visit level so that patients with multiple visits could contribute each visit to the study period. Antibiotics that were included were limited to oral agents that would plausibly be prescribed for RTDs; antibiotics administered via alternative routes (eg, topical) or exclusively used for diagnoses not related to the respiratory tract (eg, fosfomycin) were excluded, as were antiviral and antifungal agents.

To determine the appropriateness of an antibiotic prescription, we adapted a tiered appropriateness assessment strategy, originally outlined by Fleming-Dutra et al [8] and validated in the UPHS PC practices by Degnan et al 9]. In this approach, encounters were assigned to 1 of 3 tiers based on how frequently antibiotic therapy would be justified for a particular diagnosis, as assessed by ICD-10-CM codes assigned at the encounter. Tier 1 included diagnoses for which antibiotic prescribing is almost always appropriate (eg, bacterial pneumonia); tier 2 included diagnoses for which antibiotics are sometimes appropriate (eg, sinusitis); and tier 3 included diagnoses for which antibiotics are rarely indicated (eg, asthma, viral upper respiratory tract infection). For encounters in which multiple ICD-10-CM codes fell into multiple tiers of appropriateness, the lowest (ie, most appropriate) tier was selected. Visits were also grouped by the presence of ICD-10-CM codes for specific RTDs and respiratory signs and symptoms.

### Statistical Analyses

At the encounter level, patient-specific variables were compared during all 3 intervention time periods using χ^2^ or Wilcoxon rank sum testing as appropriate. A multivariable mixed-effects logistic regression model was conducted on the visit level to compare the odds of antibiotic prescribing in the post-intervention period to the prior 2 periods. We considered variables with an odds ratio (OR) having a *P* value <.25 on the univariable analysis for inclusion in the final model, adding those variables of interest one at a time and including those that were significant (*P* < .05) in the final model. To account for the interaction between the intervention and diagnosis tier seen in the initial study analysis, the model with the final set of covariates, adding a tier-intervention interaction term was assessed. Month and year were included in the model to account for seasonal variations and secular trends in antibiotic prescribing. By performing nested clustering on patient and prescriber, we accounted for the possibility of patients and prescribers contributing multiple visits during each intervention period.

Rates of antibiotic prescribing were compared using the χ^2^ test to assess rates of overall prescribing, prescribing rates among tiers of appropriateness, and prescribing rates for specific RTDs. A 2-tailed *P* value of .05 was used as the cutoff for statistical significance. All calculations were performed using Stata, version 17.0 (StataCorp, College Station, TX), with visualizations produced using R version 4.2.2 (R Foundation for Statistical Computing, Vienna, Austria) [17].

## RESULTS

There were 260 900 unique RTD encounters included in the final analysis. These encounters represented approximately 24.9% of all in-person visits during the pre-intervention period, 22.9% during the intervention period, and 23.9% during the post-intervention period. The post-intervention period comprised 75 145 of these encounters, representing 49 847 unique patients, 29 unique clinics, and 165 unique prescribers, with a median of 1 encounter per patient (interquartile range, 1–2). The initial analysis included 31 clinics; however, 2 clinics dissolved during the study period, leaving 29 PC clinics in our final analysis. Of the 167 prescribers who participated in the educational session (and were included in the initial analysis), 151 (90.4%) had at least 1 encounter in the final month of the analysis. Patient demographics are outlined in Table 1, with prescriber and clinic characteristics provided in Table 2.

**Table 1.** Patient Demographics and Encounter Characteristics

Variable	Pre-Intervention (n = 127 324)		Intervention (n = 58 431)		Post-Intervention (n = 75 145)	
Age, median (interquartile range), y	54	(39–66)	57	(41–68)	57	(41–69)
Patient gender, n (%)						
Male	44 194	(34.7)	20 547	(35.2)	26 545	(35.3)
Female	83 130	(65.3)	37 884	(64.8)	48 600	(64.7)
Patient race, n (%)						
White	87 710	(68.9)	39 638	(67.8)	51 864	(69.0)
Black or African-American	28 443	(22.3)	13 338	(22.8)	16 766	(22.3)
Asian	3308	(2.6)	1482	(2.5)	1720	(2.3)
Native Hawaiian or other Pacific Islander	242	(0.2)	111	(0.2)	91	(0.1)
American Indian or Alaskan Native	70	(0.1)	39	(0.1)	66	(0.1)
Other	3543	(2.8)	1614	(2.8)	3000	(4.0)
Unknown	4008	(3.1)	2209	(3.8)	1638	(2.2)
Patient ethnicity, n (%)						
Non-Hispanic Non-Latino	118 225	(96.5)	53 586	(95.8)	72 317	(96.2)
Hispanic Latino	4319	(3.5)	2372	(4.2)	2828	(3.8)
Lowest tier in a given encounter, n (%)						
1, always	2875	(2.3)	1526	(2.6)	2416	(3.2)
2, sometimes	33 700	(26.5)	12 385	(21.2)	18 116	(24.1)
3, rarely	90 749	(71.3)	44 520	(76.2)	54 613	(72.7)
Specific RTD diagnoses, n (%)^a^						
Sinusitis	23 381	(18.4)	7500	(12.8)	12 195	(16.2)
Acute bronchitis	9088	(7.1)	3092	(5.3)	4655	(6.2)
Pertussis	22	(0.0)	20	(0.0)	16	(0.0)
Pharyngitis	8903	(7.0)	3849	(6.6)	5276	(7.0)
Pneumonia	2730	(2.1)	1432	(2.5)	1718	(2.3)
Otitis media	6690	(5.3)	3172	(5.4)	3787	(5.0)
Respiratory tract signs/Symptoms (eg, cough, dyspnea)	36 573	(28.7)	18 316	(31.3)	24 279	(32.3)
Chronic asthma/COPD	27 804	(21.8)	14 274	(24.4)	16 737	(22.3)
Asthma exacerbation	5333	(4.2)	2718	(4.7)	3424	(4.6)
COPD exacerbation	1084	(0.9)	632	(1.1)	860	(1.1)
Allergy disorders	17 074	(13.4)	8130	(13.9)	8779	(11.7)
Viral RTD (including influenza)	1094	(0.9)	859	(1.5)	1475	(2.0)
Other acute upper respiratory tract infections^b^	10 617	(8.3)	4592	(7.9)	9093	(12.1)
Other	9049	(7.1)	3881	(6.6)	6096	(8.1)

Abbreviations: COPD, chronic obstructive pulmonary disease; RTD, respiratory tract diagnoses.

^a^An individual encounter may be associated with multiple *International Classification of Diseases, Tenth Revision, Clinical Modification*, codes and multiple RTD categories.

^b^Includes diagnoses such as tonsilitis, laryngitis, tracheitis, and unspecified acute upper respiratory tract infections.

**Table 2.** Prescriber and Clinic Characteristics

Prescriber and Clinic Characteristics (n = 179)	
Prescriber gender,%	
Male	39.4
Female	60.6
Prescriber specialty, %	
Internal medicine	46.7
Family medicine	53.3
Teaching practice, %	
Teaching	14.2
Nonteaching	85.8
Prescriber type, %	
Medical doctor/Doctor of osteopathic medicine	71.7
Nurse practitioner/Physician assistant	28.3
Practice location, %	
Urban	37.1
Nonurban	62. 9
Median years since certification (interquartile range)	14 (7–25)

**Figure 1.**
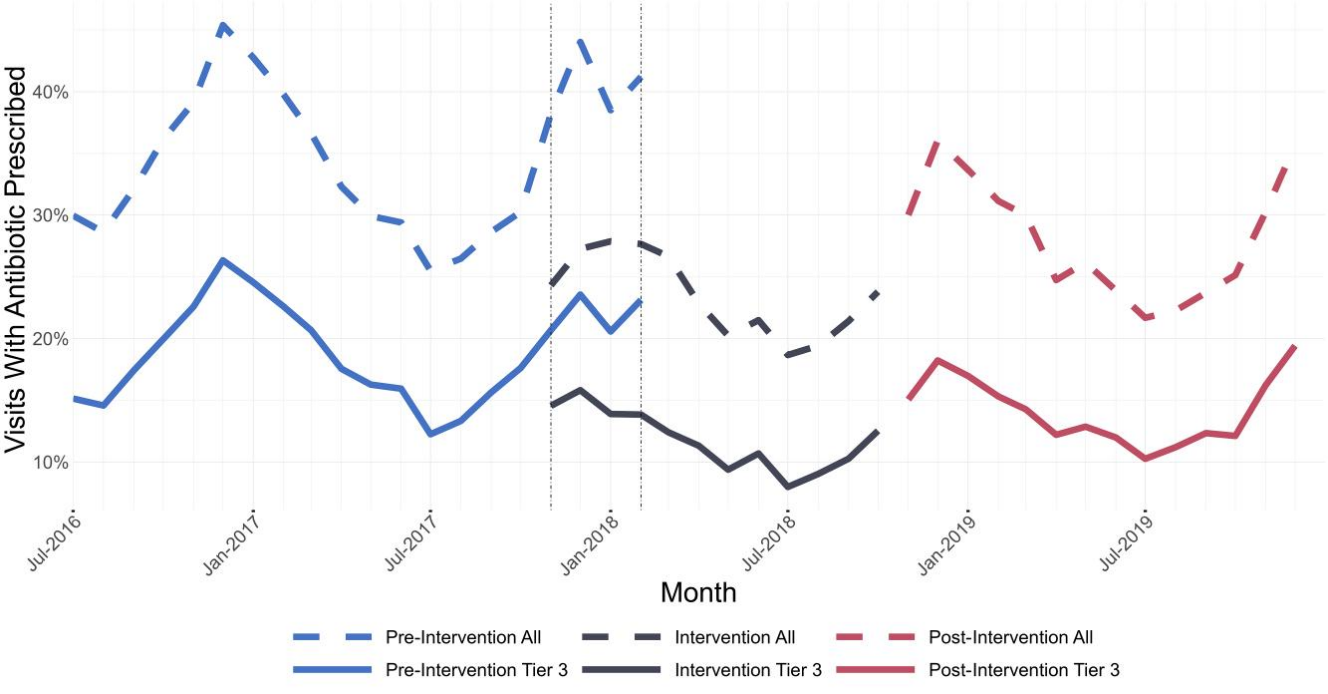
Monthly rates of antibiotic prescribing across intervention periods. Vertical dashed lines represent stepped-wise transition.

When compared with the pre-intervention period, the overall proportion of RTD visits with an antibiotic prescription was lower in the post-intervention period, with 28.9% of visits resulting in an antibiotic prescription (post-intervention) compared with 35.2% (pre-intervention); however, this rate was higher than the intervention period prescribing rate of 23.0% (*P* < .001). In tier 3 (rarely) visits, 14.4% of encounters resulted in an antibiotic prescription in the post-intervention time period compared with 19.2% (pre-intervention) and 11.3% (intervention period; *P* < .001). The change in tier 3 (rarely) prescribing between the intervention and post-intervention periods was not associated with the amount of time a given cohort of providers was exposed to the initial intervention. Rates of overall and tier 3 (rarely) encounter antibiotic prescribing are displayed in Figure 1. As RTD visits can be associated with multiple ICD-10-CM codes, multiple RTD groups may be accounted for in a single encounter, as shown in Table 3. Most notably, there was an increase in prescribing for otitis media in the post-intervention period compared with both the intervention and pre-intervention periods (55.6% vs 44.6% intervention and 50.3% pre-intervention, *P* < .001). Prescribing rates of specific antibiotics (as a proportion of total antibiotic prescriptions for each period), outlined in Table 4, showed a relative decrease in fluoroquinolone and increase in doxycycline prescriptions between the intervention and post-intervention periods.

**Table 3.** Rates of Antibiotic Prescribing for Specific Respiratory Tract Diagnosis Groups by Study Period

Variable	Pre-Intervention Antibiotic Prescription (%)	Intervention Antibiotic Prescription (%)	Post-Intervention Antibiotic Prescription (%)	Risk Ratio (Post- vs Pre-Intervention)	*P* Value^a^
Bronchitis (16 835)	6839 (75.3)	1532 (49.6)	2898 (62.3)	0.83 (.81–0.85)	<.001
Sinusitis (43 076)	20 410 (87.3)	5689 (75.9)	9848 (80.8)	0.93 (0.92–0.93)	<.001
Pharyngitis (18 028)	4637 (52.1)	1346 (35.0)	2324 (44.1)	0.85 (0.82–0.88)	<.001
Pneumonia (5880)	1248 (45.7)	597 (41.7)	728 (42.4)	0.93 (0.87–0.99)	.018
Otitis media (13 649)	3365 (50.3)	1414 (44.6)	2115 (55.6)	1.11 (1.07–1.15)	<.001
Pertussis (58)	14 (63.6)	14 (70.0)	11 (68.8)	1.08 (0.68–1.71)	.898
Respiratory tract signs/Symptoms (79 168)	9989 (27.3)	3456 (18.9)	5375 (22.1)	0.81 (0.79–0.83)	<.001
Chronic asthma/COPD (58 815)	2607 (9.9)	1135 (8.0)	1498 (9.0)	0.90 (0.84–0.96)	<.001
Asthma exacerbation (11 475)	2011 (37.7)	790 (29.1)	1132 (33.1)	0.88 (0.83–0.93)	<.001
COPD exacerbation (2576)	461 (42.5)	274 (43.4)	341 (39.7)	0.93 (0.83–1.04)	.287
Allergy disorders (33 983)	1606 (9.4)	523 (6.4)	618 (7.0)	0.75 (0.68–0.82)	<.001
Viral respiratory tract diagnoses (including influenza, 3428)	155 (14.2)	72 (8.4)	132 (8.9)	0.63 (0.51–0.79)	<.001
Other acute upper respiratory tract infections (24 302)^b^	4014 (37.8)	741 (16.1)	1695 (18.6)	0.49 (0.46–0.51)	<.001
Other (19 026)	3508 (38.8)	1041 (26.8)	2075 (34.0)	0.63 (0.61–0.66)	<.001

An individual patient encounter may be associated with multiple *International Classification of Diseases, Tenth Revision, Clinical Modification*, codes and multiple RTD categories.

Abbreviation: COPD, chronic obstructive pulmonary disease.

^a^ *P* value for χ^2^ comparison of prescribing frequency between 3 intervention periods.

^b^Includes diagnoses such as tonsilitis, laryngitis, tracheitis, and unspecified acute upper respiratory tract infection.

**Table 4.** Rates of Specific Antibiotic Prescribing by Study Period

Variable	Pre-Intervention (n = 44 786)^a^	Intervention (n = 13 457)^a^	Post-Intervention (n = 21 696)^a^	*P* Value
Azithromycin (%)	17 062 (37.0)	4445 (31.8)	6937 (31.6)	<.001
Amoxicillin-clavulanate (%)	12 024 (26.1)	4163 (29.8)	6809 (31.1)	<.001
Amoxicillin (%)	4112 (8.9)	1342 (9.6)	2045 (9.3)	.419
Doxycycline (%)	3541 (7.7)	1346 (9.6)	2965 (13.5)	<.001
Levofloxacin (%)	3420 (7.4)	1023 (7.3)	1198 (5.5)	<.001
Other (%)	5951 (12.9)	1657 (11.9)	1953 (8.9)	<.001

^a^n denotes visits with antibiotics prescribed, not total visits.

The results of the univariable and multivariable analyses are contained in Tables 5 and 6. ORs comparing the odds of prescribing during each time period and for each tier are presented separately, given that the interaction between prescribing tier and time period was found to be significant. Comparing the post-intervention period to the pre-intervention period, the odds of antibiotic prescribing were lower for both tier 3 (OR, 0.68; 95% confidence interval [CI]: .61–.75) and tier 2 (OR, 0.66; 95% CI: .59–.73), with increased odds of prescribing for tier 1 visits (OR, 1.55; 95% CI: 1.31–1.82). When comparing the post-intervention period to the intervention period, odds of antibiotic prescribing were higher for all 3 tiers of appropriateness (tier 3 OR, 1.2; 95% CI: 1.12–1.30; tier 2 OR, 1.19; 95% CI: 1.10–1.30; tier 1 OR, 1.57; 95% CI: 1.32–1.86).

**Table 5.** Univariable Analysis of Factors Associated With Antibiotic Prescribing

Variable	Odds Ratio	95% Confidence Interval	*P* Value
Intervention			
Pre-intervention	ref		
Intervention	0.48	.47–.50	<.001
Post-intervention	0.70	.68–.72	<.001
Lowest tier per encounter			
1, always	ref		
2, sometimes	2.79	2.61–2.98	<.001
3, rarely	0.16	.15–.17	<.001
Practice location			
Urban	ref		
Nonurban	1.27	1.09–1.49	.0021
Practice type			
Teaching	ref		
Nonteaching	4.52	3.39–6.01	<.001
Provider type			
Medical doctor/Doctor of osteopathic medicine	ref		
Nurse practitioner/Physician assistant	2.30	1.57–3.36	<.001
Specialty			
Internal medicine	ref		
Family medicine	2.99	2.21–4.05	<.001
Years in practice			
>7	ref		
7–13	0.65	0.61–0.70	<.001
14–24	0.45	0.40–0.50	<.001
≥25	0.41	0.34–0.49	<.001
Provider gender			
Male	ref		
Female	1.42	1.01–2.00	.0433
Patient age, y			
18–39	ref		
40–55	1.01	0.98–1.04	.4909
56–67	0.88	0.85–0.91	<.001
≥68	0.60	0.58–0.63	<.001
Patient gender			
Male	ref		
Female	1.16	1.13–1.20	<.001
Patient race			
White	ref		
Black or African-American	0.68	0.65–0.71	<.001
Asian	0.72	0.67–0.78	<.001
Native Hawaiian or other Pacific Islander	1.20	0.91–1.58	.2068
American Indian or Alaskan Native	0.89	0.56–1.43	.6304
Other	0.90	0.84–0.96	.0024
Unknown	0.82	0.77–0.88	<.001
Charlson comorbidity index, binned results			
0	ref		
≥1	0.54	0.53–0.55	<.001
Month of encounter			
Aug	ref		
Sept	1.19	1.12–1.26	<.001
Oct	1.31	1.24–1.38	<.001
Nov	1.76	1.67–1.85	<.001
Dec	2.26	2.14–2.38	<.001
Jan	1.94	1.84–2.04	<.001
Feb	1.64	1.56–1.74	<.001
Mar	1.53	1.44–1.61	<.001
Apr	1.12	1.06–1.18	<.001
May	1.04	0.98–1.10	.2103
June	1.06	0.99–1.12	.0804
July	1.03	0.97–1.09	.3370
Year of encounter			
2016	ref		
2017	0.86	0.83–0.89	<.001
2018	0.52	0.50–0.54	<.001
2019	0.61	0.59–0.63	<.001

**Table 6.** Multivariable Analysis of Factors Associated With Antibiotic Prescribing

Visit Tier	Percentage of Visits With an Antibiotic Prescribed		Adjusted Odds Ratio	95% Confidence Interval
Post-intervention vs pre-intervention period				
	Pre-intervention (%)	Post-intervention (%)		
Tier 1, always	46.3	54.7	1.55	1.31–1.82
Tier 2, sometimes	77.3	69.0	0.66	.59–.73
Tier 3, rarely	19.8	14.4	0.68	.61–.75
Post-intervention vs intervention period				
	Intervention (%)	Post-intervention (%)		
Tier 1, always	42.4	54.7	1.57	1.32–1.86
Tier 2, sometimes	62.9	69.0	1.19	1.10–1.30
Tier 3, rarely	11.3	14.4	1.20	1.12–1.30

Additional variables included in multivariable model: practice type (teaching vs nonteaching), practice specialty (family medicine vs internal medicine), provider type (medical doctor/doctor of osteopathic medicine vs nurse practitioner/physician assistant), encounter month, encounter year, Charlson comorbidity score, patient age, patient race, provider gender.

## DISCUSSION

Our analysis showed a moderately sustained reduction in the rates of both overall and tier 3 (rarely) antibiotic prescribing for RTDs after cessation of peer comparison feedback. This study adds to the growing literature surrounding efforts in outpatient antibiotic stewardship, showing that targeted interventions can have long-term effects on prescribing, though sustainability remains a challenge. While a retrospective analysis is limited in its ability to draw causal conclusions, there are a few possible attributes of the intervention that may have contributed to durability. The initial intervention was focused on RTDs rather than all outpatient antibiotics and provided evidenced-based resources that could be easily used during encounters when peer comparison feedback ceased. The provided feedback was also specific, focusing on 2 easily interpreted metrics rather than an extensive review of prescribing practices. The simplicity of feedback reporting coupled with the relatively high frequency of feedback (eg, monthly during the intervention) may have helped reinforce appropriate prescribing decisions once feedback concluded. Additionally, increasing national awareness of excessive outpatient antibiotic use may have increased the efficacy of the intervention, making prescribers more receptive to stewardship efforts [18]. However, despite the lower rates of antibiotic prescribing compared with the pre-intervention period, the increase compared with the intervention period suggests some reversion to less appropriate prescribing and may demonstrate the need for maintenance of this type of stewardship intervention to fully sustain the effects.

Gerber and colleagues reported on the long-term durability of an education and feedback program on the rates of broad-spectrum antibiotic prescribing for pediatric outpatient acute respiratory tract infections [19, 20]. While their initial intervention demonstrated a 50% reduction in broad-spectrum antibiotic prescribing, their 18-month difference-in-differences analysis showed no difference between the intervention and control groups by the end of the 12-month feedback-free period. However, a Cochrane review conducted by Arnold and Straus in 2005 of 39 trials found that long-term durability, evaluated in 3 studies, did show a sustained effect in the following 1 to 4 years [19]. In contrast to the study published by Gerber, the feedback provided in our initial study was simpler, focusing on 2 specific metrics (overall and inappropriate antibiotic prescribing), provided monthly (rather than quarterly) to prescribers. We suspect that the more targeted and repetitive nature of our intervention may have helped shift prescribers’ practice patterns. Additionally, while a longer-term retrospective analysis beyond 14 months may have shown further increases in prescribing with time, the onset of the COVID-19 pandemic limited our ability to study this without confounding. Overall, the mixed results of previously published studies suggest that outpatient stewardship programs need continual monitoring for success rather than adopting a “one-size-fits-all” approach.

During the post-intervention period, there were some notable changes in prescribing trends. We found a relative increase in doxycycline prescribing with decreased levofloxacin prescribing when compared with the pre-intervention and intervention periods. This may be reflective of national trends in fluroquinolone prescribing or could be an unintended consequence of the original intervention that was intended to reduce inappropriate antibiotic prescriptions but not necessarily to impact antibiotic choice [20, 21]. It is also notable that rates of azithromycin prescribing remained lower during the post-intervention period compared with earlier periods, contrasting national trends of increased macrolide prescribing [22]. Azithromycin has been associated with high rates of inappropriate prescribing, so the decrease seen in our study could suggest a sustained change in practice patterns [23, 24]. Rates of antibiotic prescribing for specific RTD groups (eg, sinusitis) generally followed a similar pattern of a mild increase during the post-intervention period that was lower than in the pre-intervention period. One notable exception was antibiotic prescribing for otitis media, which was higher in the post-intervention period than in the preceding periods. In exploring otitis media by ICD-10-CM code, there was an increase in relative prescribing for nonsuppurative otitis media (a tier 3 indication) in the post-intervention period (29.6% compared with 12.9% intervention and 19.8% pre-intervention), which may reflect a future educational opportunity. However, seasonal variation limits the interpretation of these results, as different intervention periods varied in the number of winter vs non-winter months.

While the focus of this analysis was on the 2 metrics associated with the feedback intervention (overall and tier 3 [rarely] prescribing), we did note some trends in tier 1 (always) diagnoses and antibiotic prescribing. There was a slight increase in tier 1 diagnoses in the post-intervention period compared with prior periods (3.2% in the post-intervention period vs 2.6% in the intervention period and 2.3% in the pre-intervention period). Notably, there was a difference in the seasonal composition of the 3 intervention time periods, with a higher proportion of fall/winter months in the post-intervention period, which influences the rates of both RTDs and antibiotic prescribing. In analyzing the breakdown of ICD-10-CM codes, we noted that a majority of tier 1 (always) visits were for pneumonia diagnoses across all 3 periods. However, there was an increase in streptococcal pharyngitis during the post-intervention period (n = 591) compared with the intervention (n = 1) and pre-intervention (n = 15) periods. The increase was not limited to a single clinic or cohort of providers. The lack of provider feedback during this period makes diagnostic shifting (eg, selecting a higher tier code to justify antibiotic prescribing) unlikely. While the Centers for Disease Control and Prevention does not monitor trends in streptococcal pharyngitis, there was no increase in invasive group A *Streptococcus* infections during this time period [25, 26]. We suspect that this may be due to broader adoption of rapid antigen testing and plan to monitor this as part of ongoing outpatient stewardship efforts. While low prescribing rates for pneumonia (a tier 1 diagnosis) were noted in all 3 intervention periods, many patients with an RTD visit for pneumonia are seen in follow-up after a hospital or emergency department encounter, which was confirmed by random chart review [15].

The main strength of this study is that it builds on a wealth of validated prescribing data included in the initial trial. By using data from a large cohort of PC clinics and prescribers for whom we have already assessed the efficacy of education and peer-comparison feedback, we were able to study the post-intervention effects and decay over time when the ongoing feedback part of the intervention ended. The time studied also enhances the strength of the study, allowing us to account for seasonal variations in prescribing over the course of a year yet still concluding the analysis prior to the onset of the COVID-19 pandemic. By including both month and year in our multivariable analysis, we accounted for seasonality and secular trends that influence antibiotic prescribing.

However, as the entirety of this study was performed before the rapid expansion of telemedicine with the COVID-19 pandemic, we were unable to study associations between the intervention and rates of antibiotic prescribing for telemedicine encounters. In a meta-analysis, Suzuki and colleagues found increased rates of prescribing for certain RTDs in telemedicine encounters, which, if seen in our population, would lead to a further reduction of the impact of our intervention [27]. Additionally, our study was limited to prescribers who participated in the initial education and feedback intervention and continued to practice in the UPHS health system. This means that the cohort of providers decreased with time due to job changes and retirements and that providers who joined the health system later and were not part of the initial intervention were excluded from this analysis, a trend likely amplified during the COVID-19 pandemic. While providers not included in the initial intervention had higher rates of overall antibiotic prescribing in the post-intervention period (31.1% vs 28.9%, *P* < .001), we were limited in our ability to draw meaningful conclusions from this data. All UPHS PC providers were included in the initial intervention, meaning there was no control group, and there was minimal turnover during the intervention period, and thus data are only available for the post-intervention period. Continuing to study these trends in prescribing over time may help inform the generalizability of this type of intervention, whether it creates a “cultural shift” within a practice or is limited to only prescribers who receive an intervention. This will be an important direction for future research, as prior studies have shown that prescriber attitudes and beliefs play a significant role in antibiotic use [1, 14].

This study builds on the results of the initial trial, demonstrating that despite increased antibiotic prescribing in the absence of feedback, rates of both overall and inappropriate antibiotic prescribing remained lower early in the post-intervention period. These results support the need for ongoing interventions as part of an active outpatient antibiotic stewardship program to educate and monitor appropriate antibiotic prescribing. Additional studies are needed to determine the ideal balance between education and feedback as well as the optimal frequency of feedback (eg, monthly, quarterly, on-demand), not only to allocate stewardship resources but also to balance the administrative burdens associated with performance feedback [28]. Future directions for this work will include an examination of the impact of telemedicine on outpatient prescribing for RTDs as well as optimal methods to maintain the effects of the intervention.

## Notes

***Financial support*.** This work was supported by a Centers for Disease Control and Prevention (CDC) Cooperative Agreement Funding Opportunity Announcement CK16-004, Epicenters for the Prevention of Healthcare Associated Infections. W. B. B. reports support from the Southeastern Pennsylvania Adult and Pediatric Prevention Epicenter Network (U54-CK000610-02).
